# Supplementary material for: Filament formation drives catalysis by glutaminase enzymes important in cancer progression
Source: Nat Commun. 2024 Mar 4;15:1971. doi: 10.1038/s41467-024-46351-3 (PMC10912226; doi:10.1038/s41467-024-46351-3)
Supplement: Supplementary file 1 — Supplementary Information [file 41467_2024_46351_MOESM1_ESM.pdf]

Supplementary Information for

Filament formation drives catalysis by glutaminase enzymes important in cancer progression

Authors

Shi Feng[1], Cody Aplin[1], Thuy-Tien T. Nguyen[1], Shawn K. Milano[1], and Richard A. Cerione[1,2]*

Affiliation

[1]Department of Chemistry and Chemical Biology, Cornell University, Ithaca, New York, 14853

[2]Department of Molecular Medicine, Cornell University, Ithaca, New York, 14853

*Corresponding author: rac1@cornell.edu

This PDF contains:

Supplementary Figures 1-12

Supplementary Table 1

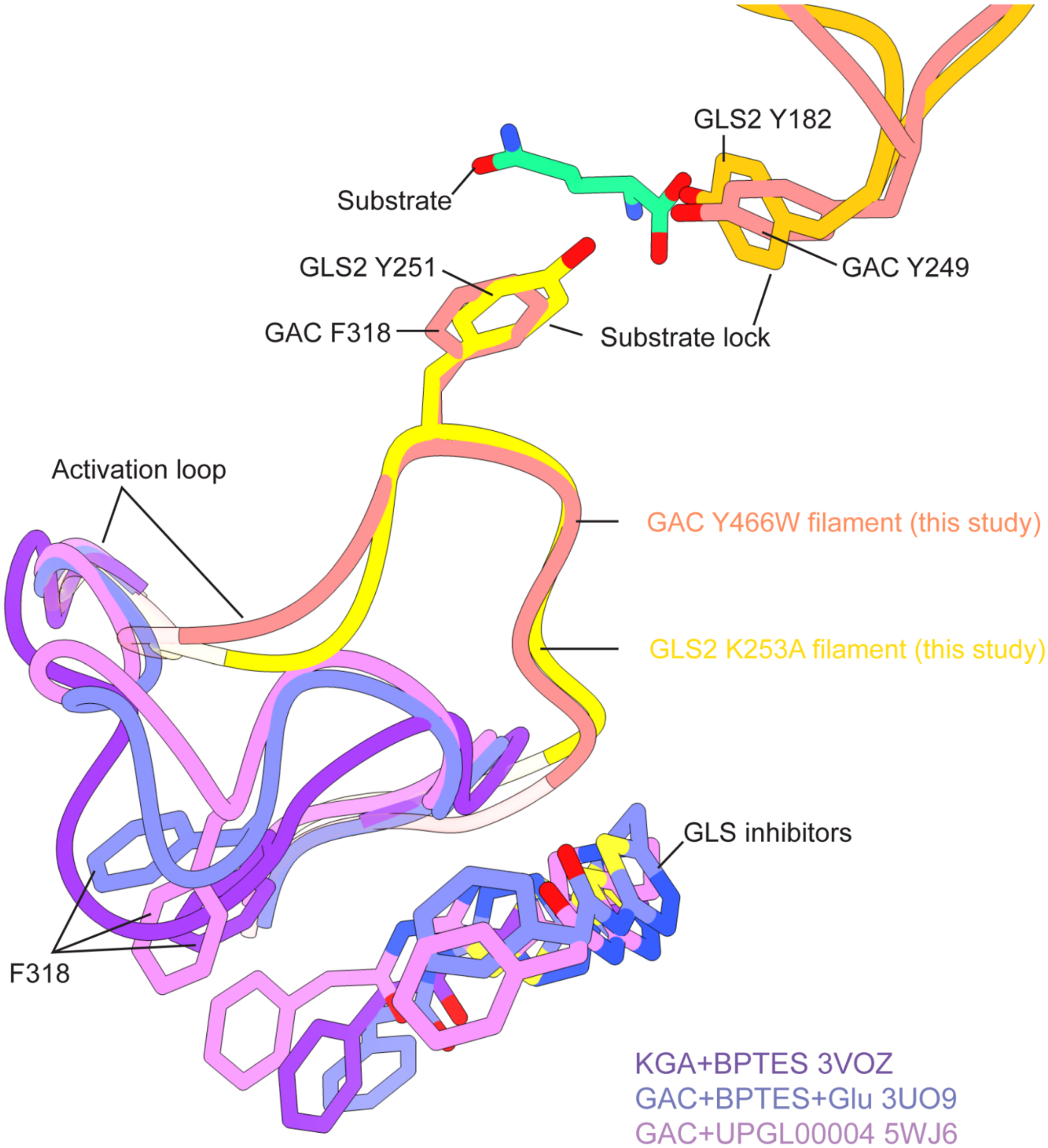

Supplementary Figure 1. The activation loops of the GAC (Y466W) filament (salmon), GLS2 (K253A) filament (yellow) and GAC bound to the allosteric inhibitors BPTES, alone or together with glutamate, and UPGL0004 (purple, lavender and pink, respectively).

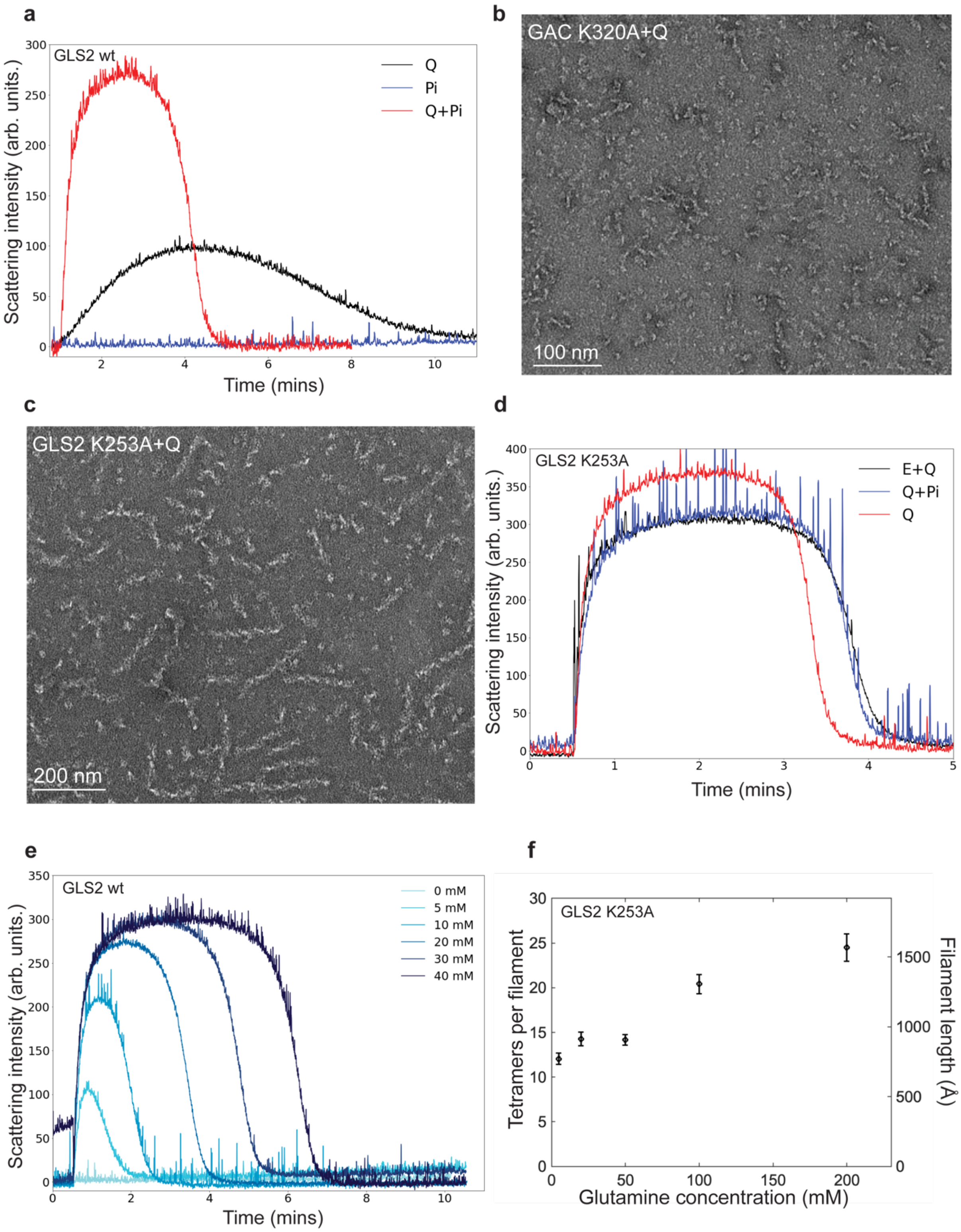
a
GLS2 wt
Q
Pi
Q+Pi
Scattering intensity (arb. units.)
Time (mins)
b
GAC K320A+Q
100 nm
c
GLS2 K253A+Q
200 nm
d
GLS2 K253A
E+Q
Q+Pi
Q
Scattering intensity (arb. units.)
Time (mins)
e
GLS2 wt
0 mM
5 mM
10 mM
20 mM
30 mM
40 mM
Scattering intensity (arb. units.)
Time (mins)
f
GLS2 K253A
Tetramers per filament
Filament length (Å)
Glutamine concentration (mM)

Supplementary Figure 2. (a) Right-angle light scattering (RALS) (n=3 independent experiments) of GLS2 (2 μM) after the addition of glutamine (Q, 20 mM), inorganic phosphate Pi (100 mM), or Q (20 mM) and Pi (100 mM). X-axis: time in minutes (mins); Y-axis: the intensity of the absorbance at 340 nm in arbitrary units (arb. units). **(b)** Negative stain EM of GAC (K320A) (1 μM) after adding Q (20 mM). **(c)** Negative stain EM of GLS2 (K253A) (1 μM) after adding Q (20 mM). **(d)** RALS (n=3 independent experiments) of GLS2 (K253A) (2 μM) after the addition of glutamate (E, 20 mM) and Q (20 mM) (black), Q (20 mM) (red), or Q (20 mM) and Pi (100 mM) (blue). X-axis: time in minutes (mins); Y-axis: the intensity of the absorbance at 340 nm in arbitrary units (arb. units). **(e)** RALS (n=3 independent experiments) of GLS2 (2 μM) with Pi (100 mM) after the addition of increasing concentrations of glutamine. X-axis: time in minutes (mins); Y-axis: the intensity of the absorbance at 340 nm in arbitrary units (arb. units). **(f)** The average number of GLS2 K253A tetramers per filament plotted against substrate (glutamine) concentration. The filament length was determined through an analysis of negative stain electron microscopy images (n=3 independent images) with ImageJ. The width of each GLS2 tetramer is 64 Å, as measured from the high resolution cryo-EM structure of the GLS2 filament. X-axis: the concentration of substrate (glutamine) in mM; left Y-axis: the number of tetramers per filament; right Y-axis: the average length of filaments in Å. Circles indicate the mean value, and error bars are shown for the standard error. Source data are provided as a Source Data file.

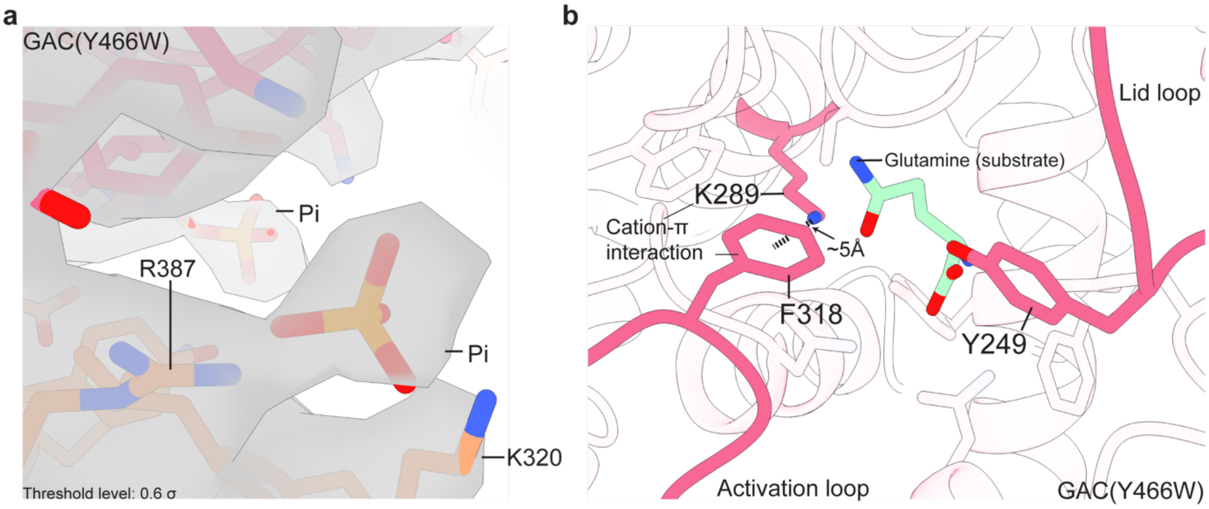

Supplementary Figure 3. (a) the electron density of inorganic phosphate (Pi) bound near the activation loop in the GAC (Y466W) filament. **(b)**: the phenylalanine-tyrosine lock in GAC consisting of Phe318 from the activation loop (magenta) and Tyr249 from the lid loop (magenta). The substrate, glutamine, is shown in green. Lys289 interacts with Phe318 from the activation loop through a cation-π interaction.

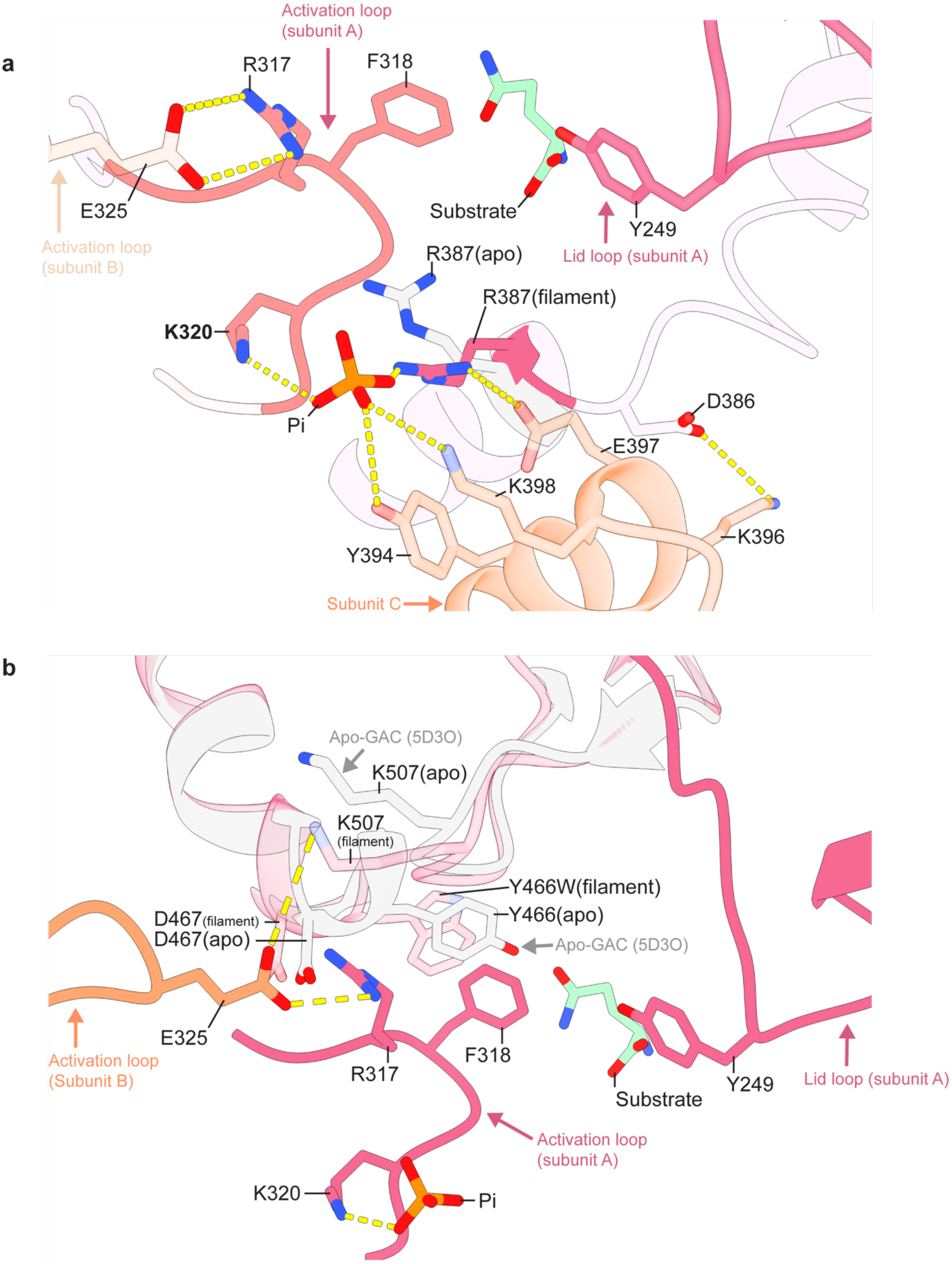
a
Activation loop
(subunit A)
R317
F318
E325
Substrate
Y249
Activation loop
(subunit B)
R387(apo)
Lid loop (subunit A)
R387(filament)
K320
D386
Pi
E397
K398
K396
Y394
Subunit C
b
Apo-GAC (5D3O)
K507(apo)
K507
(filament)
Y466W(filament)
D467(filament)
Y466(apo)
D467(apo)
Apo-GAC (5D3O)
E325
F318
Activation loop
(Subunit B)
R317
Substrate
Y249
Lid loop (subunit A)
Activation loop
(subunit A)
K320
Pi

Supplementary Figure 4. (a) The anionic activator, inorganic phosphate (Pi), interacts with Lys320 of the activation loop, and Arg387, Tyr394, and Lys398 from the dimer-dimer interface of GAC (Y466W), shown as yellow dashed lines. **(b)** The conserved residue in the active site, Tyr466, is already inserted into the active site even without the new interaction of Arg317-Asp467 and Arg317-Glu325 that occurs within the filament structure.

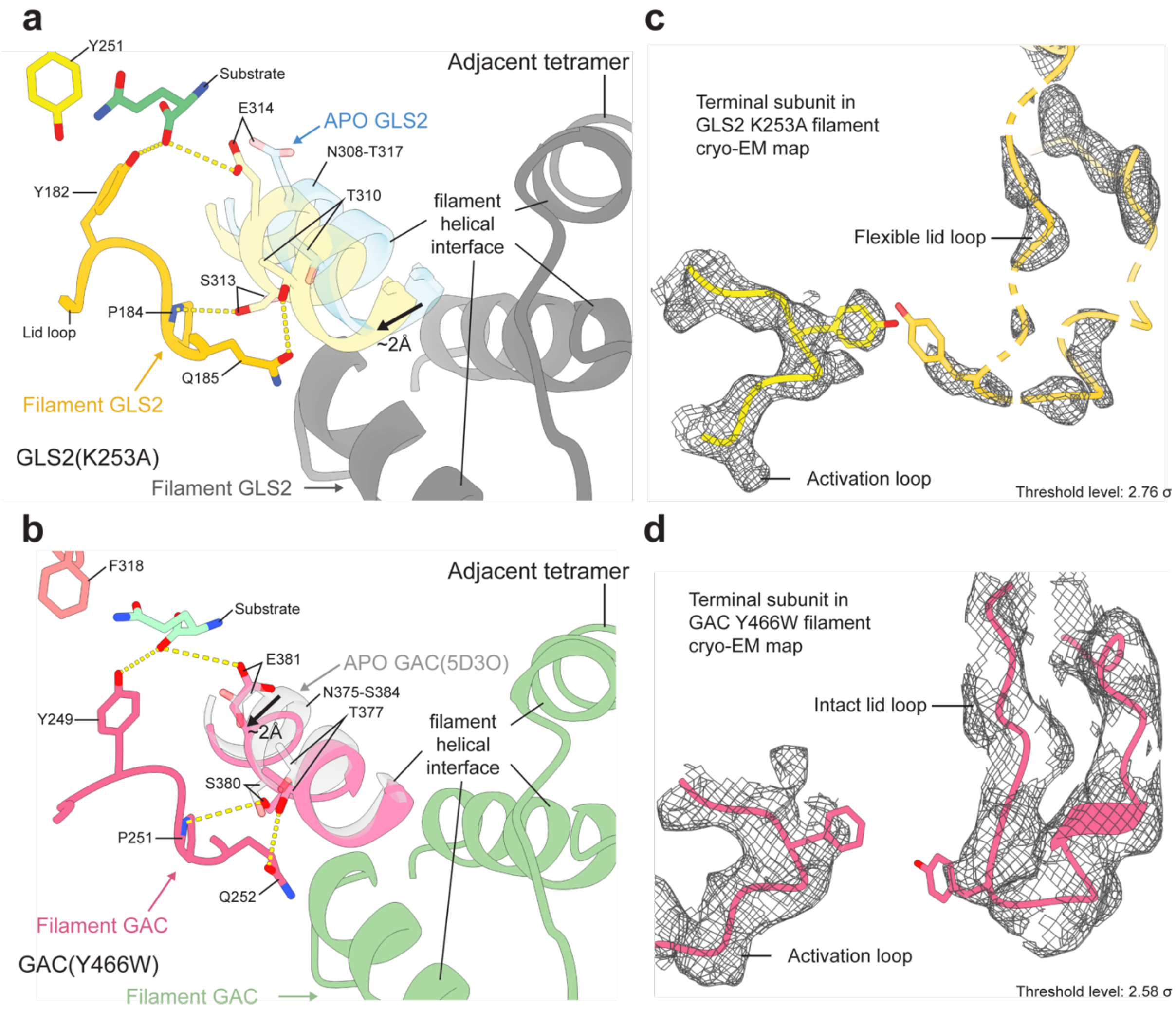

Supplementary Figure 5. (a) the lid loop of GLS2 is stabilized in the active conformation through a lid lock. Hydrogen bonds are indicated by yellow dashed lines. Apo-GLS2 is light blue and the GLS2 filament is shown in yellow and orange. The adjacent tetramer that is forming the helical interface is shown in dark gray. The arrow indicates the direction of the conformational change. **(b)** the lid loop of GAC is stabilized in the active conformation by a lid lock involving the lid loop and residues adjacent to the filament helical interface between tetramers within the filament. Hydrogen bonds are indicated by yellow dashed lines. Apo-GAC is light gray, and the GAC filament is shown in magenta. The adjacent tetramer in the filament is shown in green. The arrow indicates the direction of the conformational change. **(c)** The weak density of the lid loop in the terminal tetramer of the GLS2 filament cryo-EM map

suggests its flexibility during the catalytic turnover. **(d)** The lid loop in the catalytic-deficient GAC (Y466W) filament has strong density in the terminal subunit of the cryo-EM map.

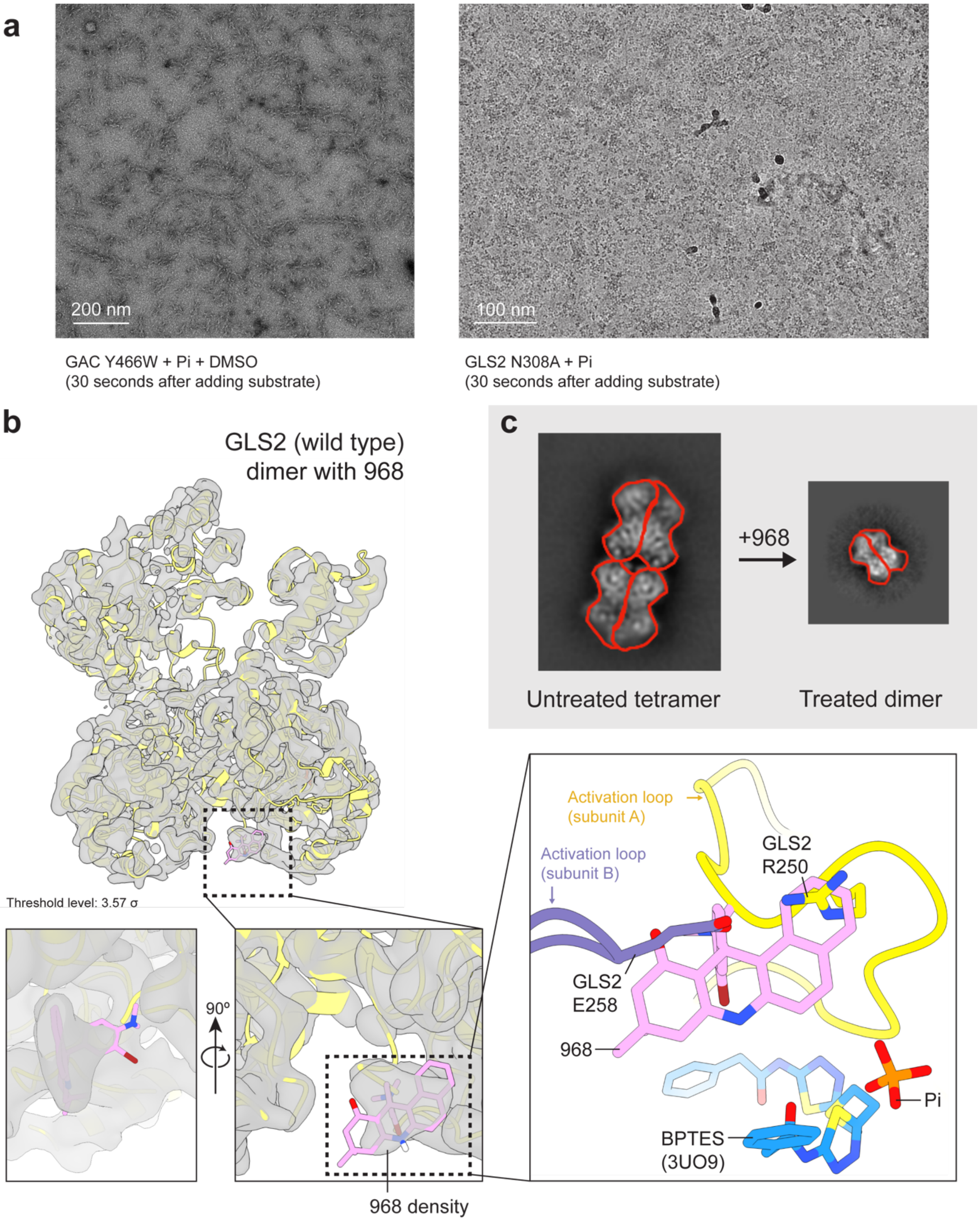
a
200 nm
100 nm
GAC Y466W + Pi + DMSO
(30 seconds after adding substrate)
GLS2 N308A + Pi
(30 seconds after adding substrate)
b
GLS2 (wild type)
dimer with 968
Threshold level: 3.57 σ
90º
968 density
c
+968
Untreated tetramer
Treated dimer
Activation loop
(subunit A)
Activation loop
(subunit B)
GLS2
R250
GLS2
E258
968
Pi
BPTES
(3UO9)

Supplementary Figure 6. (a) Left: negative stain EM (n=3 independent images) of GAC Y466W (1 μM) with inorganic phosphate (Pi, 50 mM) and DMSO (2%) 30 seconds after adding glutamine (Q, 20 mM). Right: a cryo-EM image (n=3 independent images) of GLS2 N308A (8 μM) with inorganic phosphate (Pi, 100 mM) 30 seconds after adding glutamine (Q, 20 mM). **(b)** Upper left: the 3D reconstruction of wild type GLS2 bound to 968. Apo-GLS2 model is docked. Bottom left inset: The electron density for 968 suggests that it interacts with the activation loops. Bottom right inset: The relative positions of 968, BPTES, and inorganic phosphate (Pi) are shown. Notably, 968 interferes with the interaction between Arg250 on one activation loop and Glu258 on an adjoining activation loop. GLS2 K253A filament model is docked. **(c)** cryo-EM 2D classification of GLS2 treated with 968 shows that approximately 10% of the particles can be found as dimers in the presence of 968, whereas no dimers are detected in the absence of 968. Each monomer depicting the GLS2 tetramer and dimer is outlined in red. Source data are provided as a Source Data file.

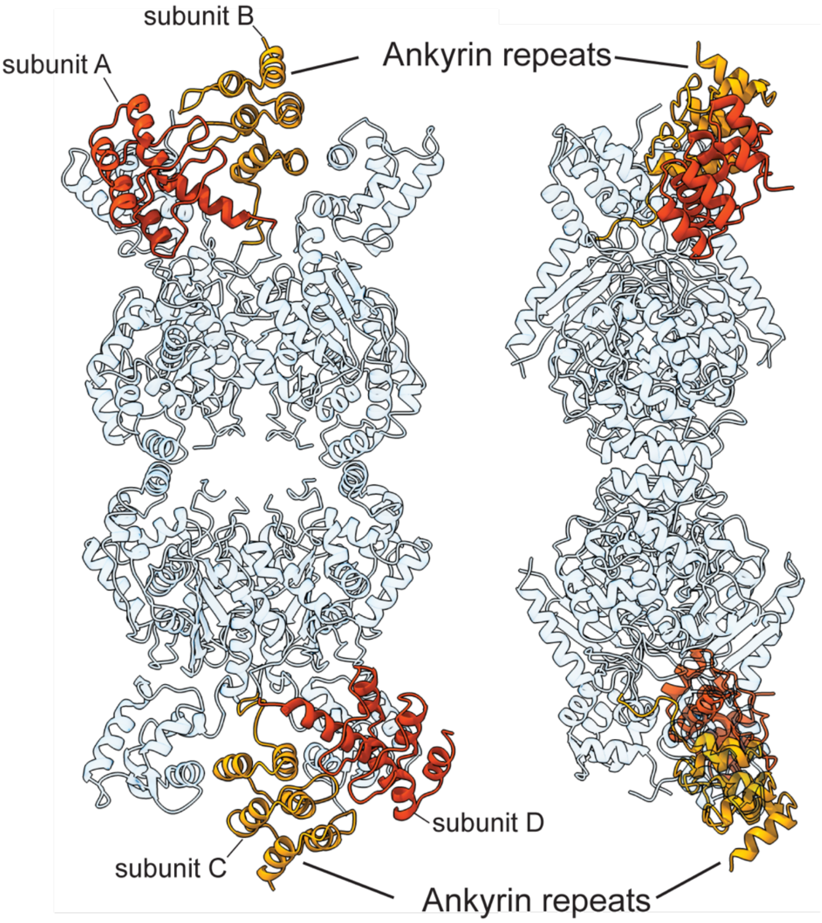

Supplementary Figure 7. The asymmetric positioning of the ankyrin repeats in apo-GLS2. Ankyrin repeats from different subunits are shown in orange and yellow and designated as subunit A, B, C, and D. Left: subunit A and B from the same dimer are asymmetrically positioned in the space between N-terminus. Subunit C and D from another dimer also display the same asymmetricity. Right: two sets of ankyrin repeats, i.e., the ankyrin repeats from subunits A/B and subunits C/D are predominantly positioned on the same side of the tetramer.

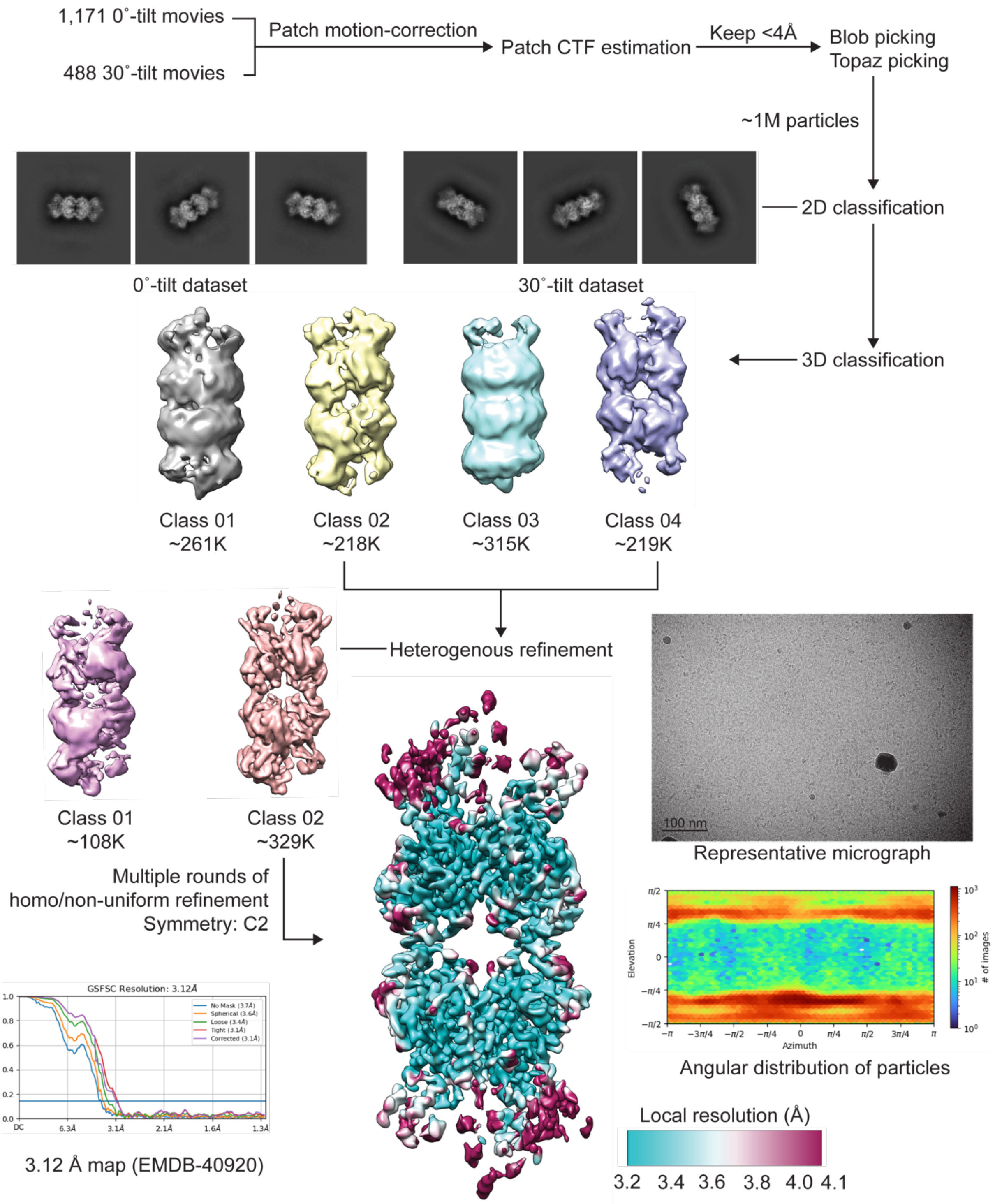

Supplementary Figure 8. The cryo-EM data processing workflow of apo-GLS2.

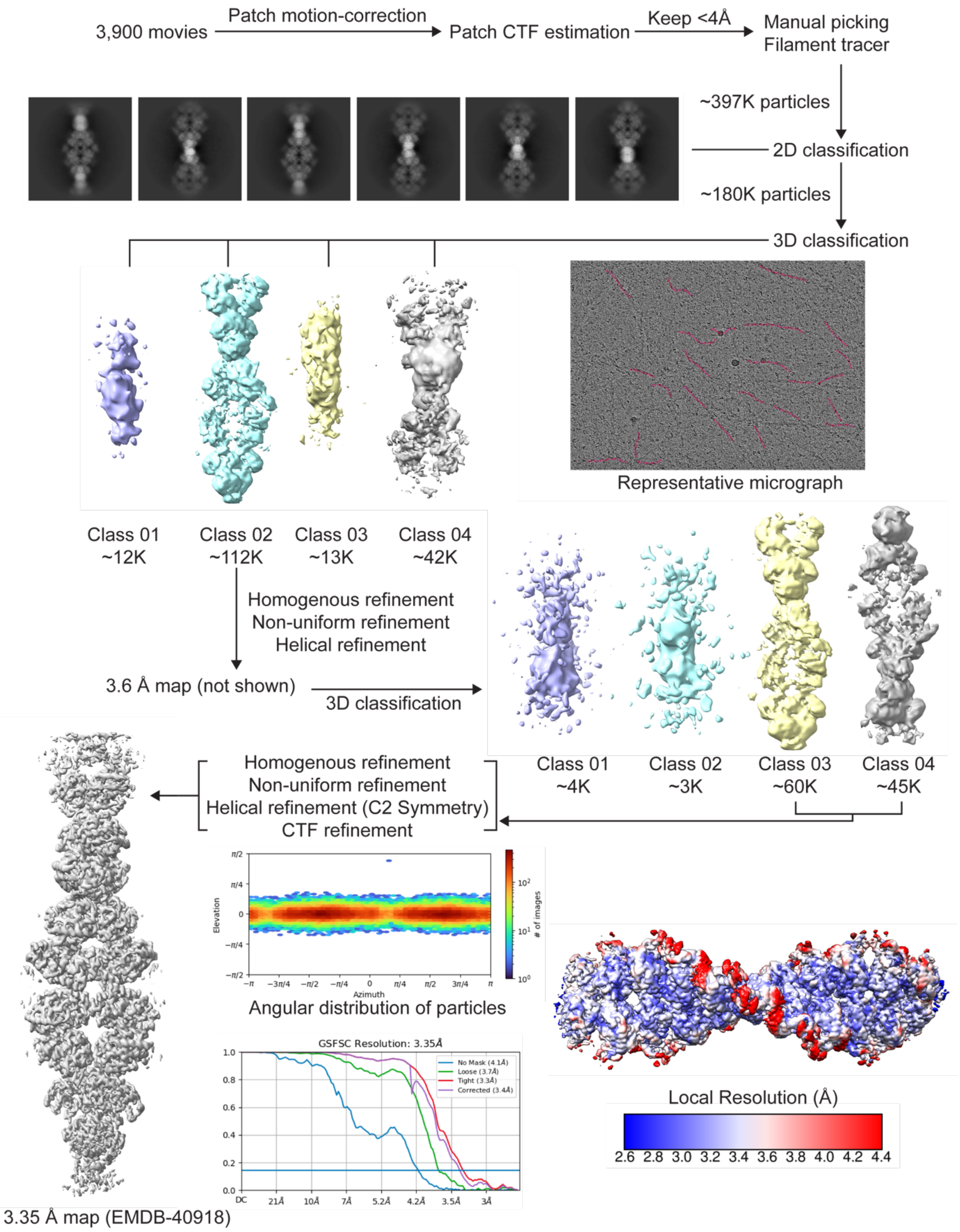

Supplementary Figure 9. The cryo-EM data processing workflow of GAC Y466W filament.

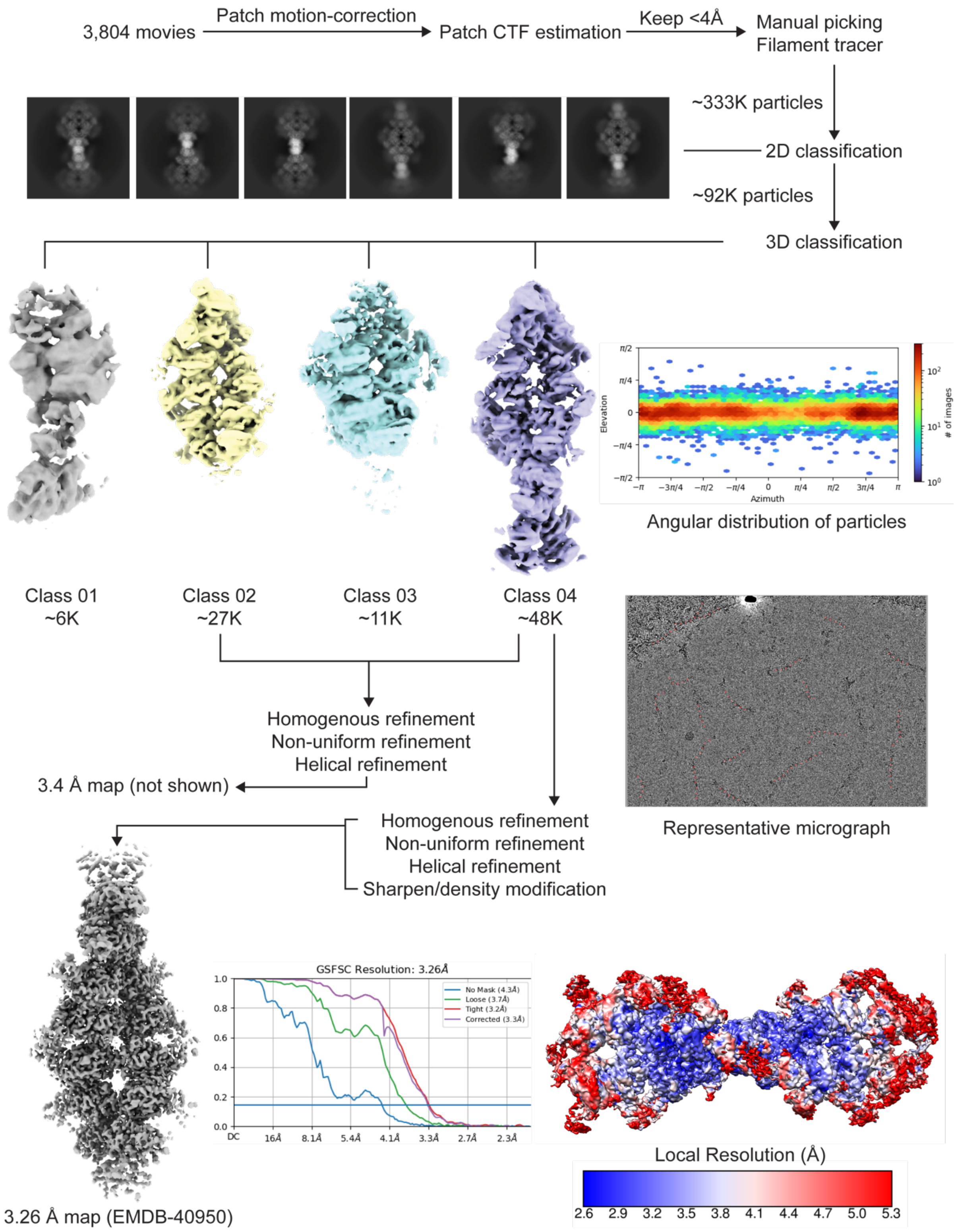

Supplementary Figure 10. The cryo-EM data processing workflow of GLS2 K253A filament.

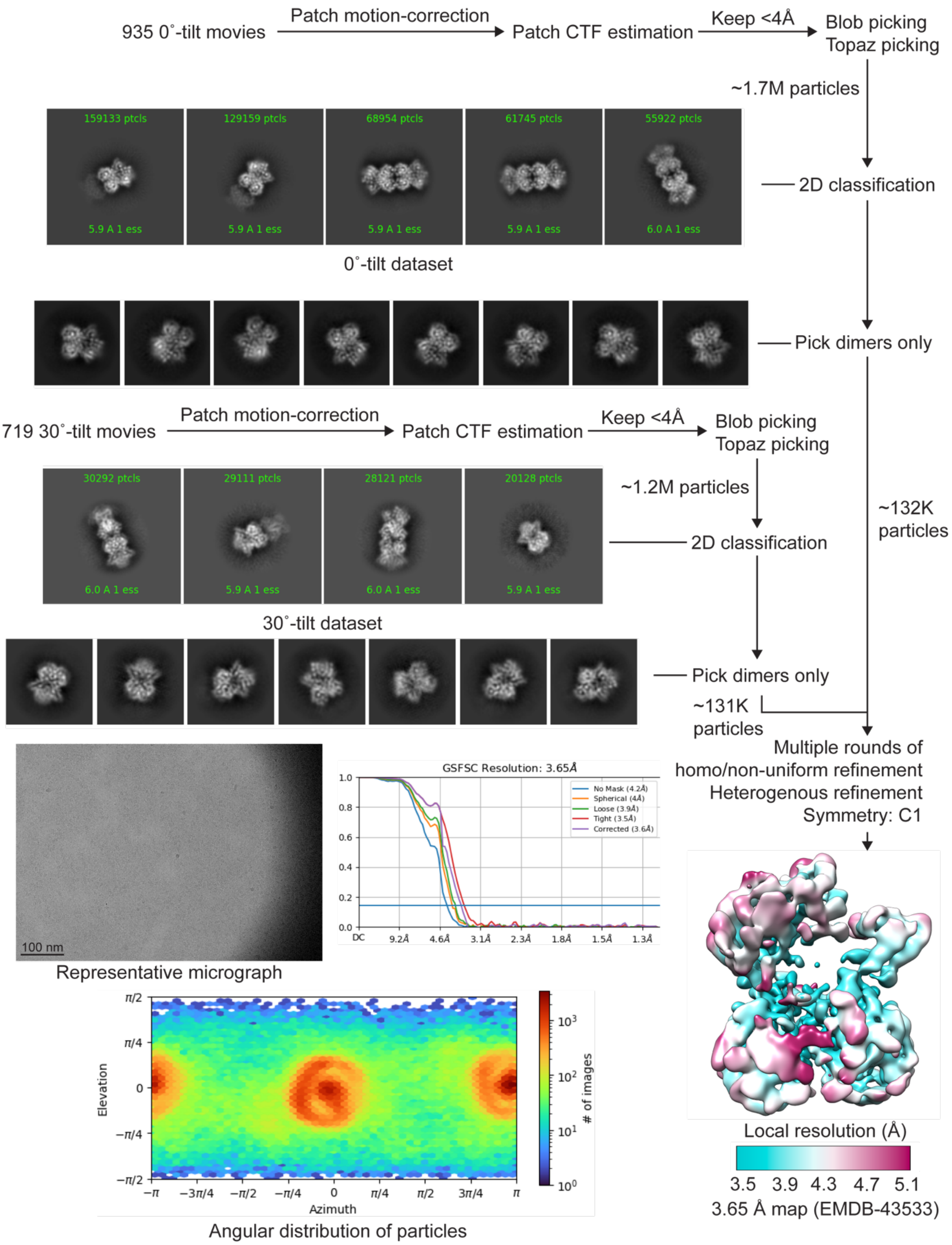

Supplementary Figure 11. The cryo-EM data processing workflow of GLS2 dimer induced by the inhibitor 968.

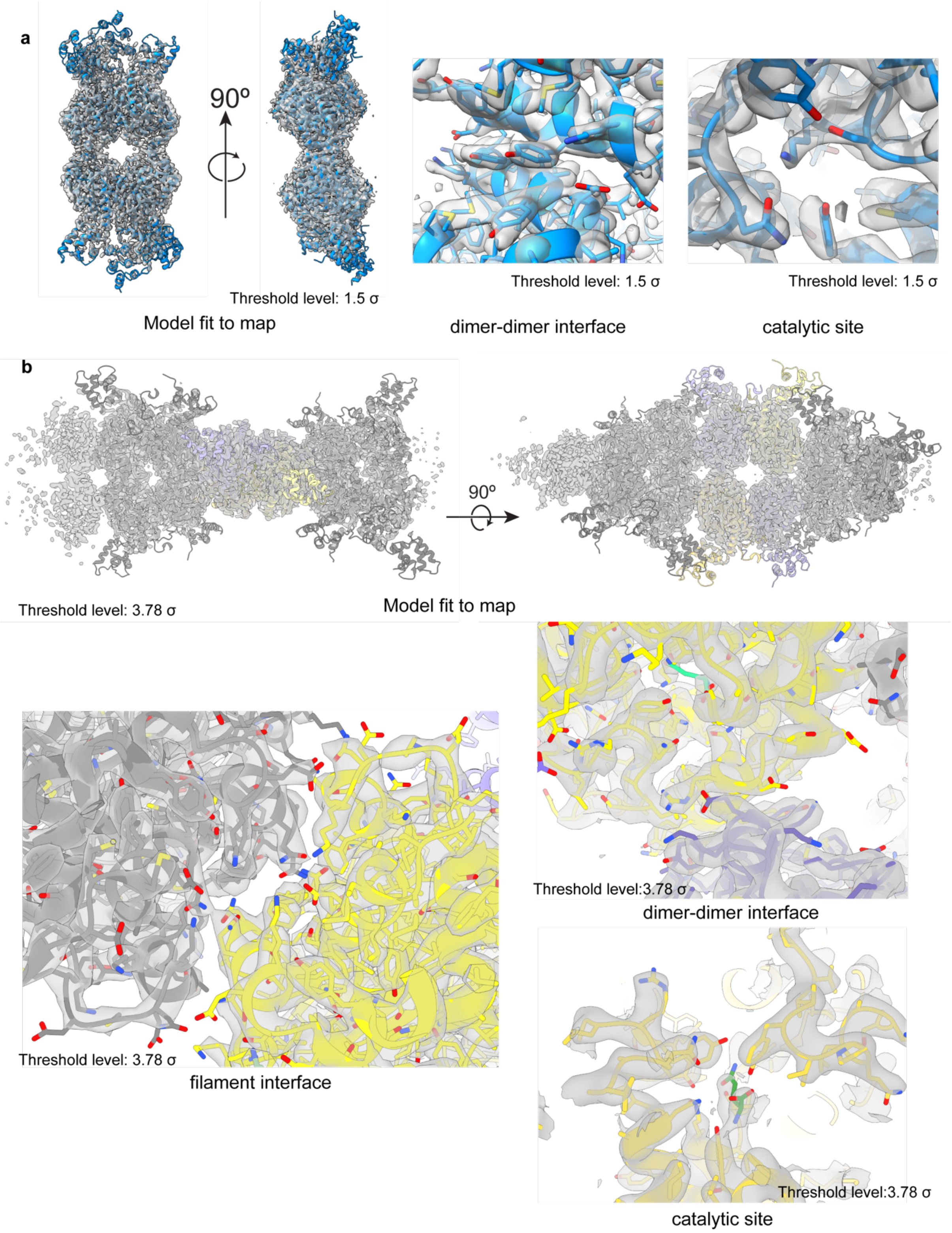

Supplementary Figure 12. The model-to-map fittings and the quality of cryo-EM maps for **(a)** apo GLS2 and **(b)** GLS2 K253A filament.

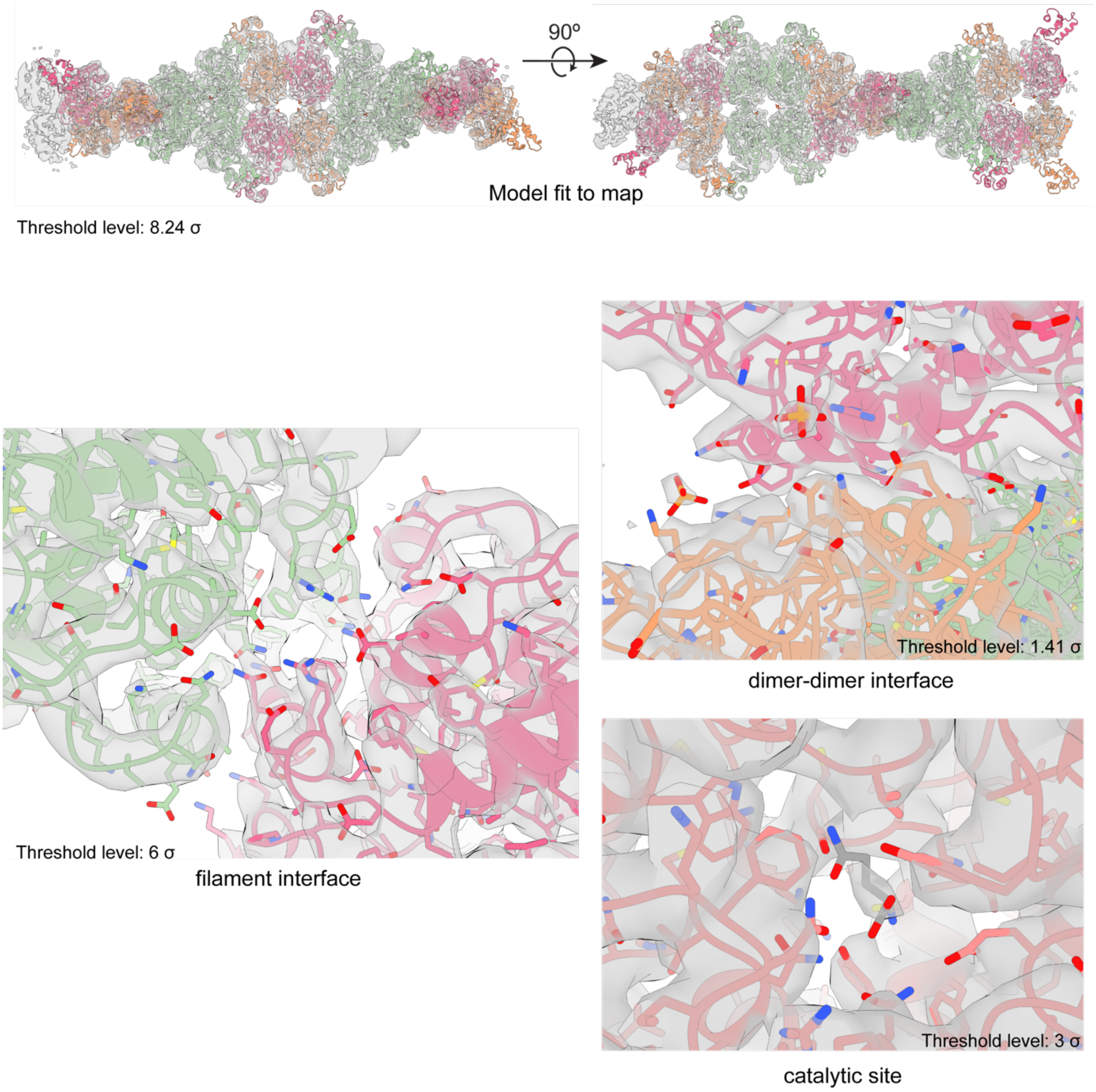

Supplementary Figure 13. The model-to-map fittings and the quality of the cryo-EM map for GAC Y466W filament.

Supplementary Table 1. Cryo-EM data collection, refinement and validation statistics.

	Apo GLS2 (EMD-40920) (PDB 8SZL)	GAC(Y466W) filament (EMD-40918) (PDB 8SZJ)	GLS2(K253A) filament (EMD-40950) (PDB 8T0Z)	968-GLS2 dimer (EMD-43533)
**Data collection and processing**				
Magnification	63kx	63kx	81kx	63kx
Voltage (kV)	200	200	300	200
Electron exposure (e–/Å^2)	50	50	50	38(0°)/48.5(30°)
Defocus range (μm)	-0.8~-2.0	-0.8~-2.0	-0.8~-2.0	-0.8~-2.0
Physical pixel size (Å)	1.23	1.31	1.058	1.23
Symmetry imposed	C2	C2	C1	C1
Initial particle images (no.)	~1M	~397K	~333K	~2.8M
Final particle images (no.)	~329K	~45K	~49K	~263K
Map resolution (Å)	3.12	3.35	3.26	3.65
FSC threshold	0.143	0.143	0.143	0.143
Map resolution range (Å)	3.0-5.0	2.9-8.3	2.2-45.5	3.5-5.5
**Refinement**				
Initial model used (PDB code)	4BQM, 5U0K	5W2J	8SZL	
Model resolution (Å)	3.12	3.35	3.26	
FSC threshold	0.143	0.143	0.143	
Model resolution range (Å)	3.0-5.0	2.9-8.3	2.2-45.5	
Map sharpening *B* factor (Å^2)	164.90	112.1	211.56	
Model composition				
Non-hydrogen atoms	15,084	36,204	38,028	
Protein residues	1,960	4,656	4,920	
Ligands	0	24	12	
B factors (Å^2)				
Protein	45.37	136.94	89.43	
Ligand	N/A	242.38	N/A	
R.m.s. deviations				
Bond lengths (Å)	0.005	0.010	0.004	
Bond angles (°)	1.021	1.110	1.031	
Validation				
MolProbity score	1.59	1.66	1.63	
Clashscore	5.47	5.27	4.75	
Poor rotamers (%)	0.00	0.00	0.02	
Ramachandran plot				
Favored (%)	95.76	94.48	94.43	
Allowed (%)	4.24	5.52	5.53	
Disallowed (%)	0.00	0.00	0.04	
