# Supplementary material for: Filament formation drives catalysis by glutaminase enzymes important in cancer progression
Source: Nat Commun. 2024 Mar 4;15:1971. doi: 10.1038/s41467-024-46351-3 (PMC10912226; doi:10.1038/s41467-024-46351-3)
Supplement: Supplementary file 3 — Description of Additional Supplementary Files [file 41467_2024_46351_MOESM3_ESM.pdf]

File name: Supplementary Movie 1

Description: The ankyrin repeats of GLS2 may hinder a scissor-like movement that is required for activation. Glutaminase undergoes a scissor-like movement to allosterically regulate the formation of salt bridge between two activation loops, which is necessary for the catalysis of glutamine. The ankyrin repeats of GLS2 may hinder the scissor-like movement, causing lower activity than that of GAC.
