# Supplementary material for: Filament formation drives catalysis by glutaminase enzymes important in cancer progression
Source: Nat Commun. 2024 Mar 4;15:1971. doi: 10.1038/s41467-024-46351-3 (PMC10912226; doi:10.1038/s41467-024-46351-3)
Supplement: Supplementary file 5 — Reporting Summary [file 41467_2024_46351_MOESM5_ESM.pdf]

nature portfolio

Corresponding author(s): Richard A. Cerione

Last updated by author(s): Jan 24, 2024

Reporting Summary

Nature Portfolio wishes to improve the reproducibility of the work that we publish. This form provides structure for consistency and transparency in reporting. For further information on Nature Portfolio policies, see our Editorial Policies and the Editorial Policy Checklist.

Statistics

For all statistical analyses, confirm that the following items are present in the figure legend, table legend, main text, or Methods section.

n/a	Confirmed	
☐	☒	The exact sample size (*n*) for each experimental group/condition, given as a discrete number and unit of measurement
☐	☒	A statement on whether measurements were taken from distinct samples or whether the same sample was measured repeatedly
☒	☐	The statistical test(s) used AND whether they are one- or two-sided<br>*Only common tests should be described solely by name; describe more complex techniques in the Methods section.*
☒	☐	A description of all covariates tested
☒	☐	A description of any assumptions or corrections, such as tests of normality and adjustment for multiple comparisons
☐	☒	A full description of the statistical parameters including central tendency (e.g. means) or other basic estimates (e.g. regression coefficient) AND variation (e.g. standard deviation) or associated estimates of uncertainty (e.g. confidence intervals)
☒	☐	For null hypothesis testing, the test statistic (e.g. F, t, r) with confidence intervals, effect sizes, degrees of freedom and P value noted<br>*Give P values as exact values whenever suitable.*
☒	☐	For Bayesian analysis, information on the choice of priors and Markov chain Monte Carlo settings
☒	☐	For hierarchical and complex designs, identification of the appropriate level for tests and full reporting of outcomes
☒	☐	Estimates of effect sizes (e.g. Cohen's *d*, Pearson's *r*), indicating how they were calculated

Our web collection on statistics for biologists contains articles on many of the points above.

Software and code

Policy information about availability of computer code

Data collection	EPU v2.10 (Thermo Fisher Scientific) and Serial EM v4.0, for Cryo-EM data acquisition
Data analysis	MotionCor 2, Relion v4.0.0, CryoSPARC v4.2.1, Topaz v0.2.3, UCSF ChimeraX v1 .4.1, ISOLDE v1 .0b3, SBGrid v2.9.9, Phenix v.1.20.1-4487, Coot v0.9.8, MolProbity v4.2, MATLAB R2022b, Matplotlib v3.7.1, SciPy 1.9.2, ImageJ v2.1.0/1.53c

For manuscripts utilizing custom algorithms or software that are central to the research but not yet described in published literature, software must be made available to editors and reviewers. We strongly encourage code deposition in a community repository (e.g. GitHub). See the Nature Portfolio guidelines for submitting code & software for further information.

Data

Policy information about availability of data

All manuscripts must include a data availability statement. This statement should provide the following information, where applicable:

- Accession codes, unique identifiers, or web links for publicly available datasets
- A description of any restrictions on data availability
- For clinical datasets or third party data, please ensure that the statement adheres to our policy

The cryo-EM density maps and the atomic model for apo-GLS2, GAC (Y466W) and the GLS2 (K253A) filament structure reported in this paper have been deposited in EM Data Bank (EMDB: EMD-40920, EMD-40918, and EMD-40950) and the Protein Data Bank (PDB: 8SZL, 8SZJ, and 8T0Z), respectively. Source data are provided with this paper, as well as on Figshare (10.6084/m9.figshare.25055630).

Research involving human participants, their data, or biological material

Policy information about studies with human participants or human data. See also policy information about sex, gender (identity/presentation), and sexual orientation and race, ethnicity and racism.

Reporting on sex and gender	N/A
Reporting on race, ethnicity, or other socially relevant groupings	N/A
Population characteristics	N/A
Recruitment	N/A
Ethics oversight	N/A

Note that full information on the approval of the study protocol must also be provided in the manuscript.

Field-specific reporting

Please select the one below that is the best fit for your research. If you are not sure, read the appropriate sections before making your selection.

☒ Life sciences ☐ Behavioural & social sciences ☐ Ecological, evolutionary & environmental sciences

For a reference copy of the document with all sections, see nature.com/documents/nr-reporting-summary-flat.pdf

Life sciences study design

All studies must disclose on these points even when the disclosure is negative.

Sample size	For cryo-EM data, no statistical methods were used to estimate the initial sample size (particle numbers). The size of particles dataset is determined when the final resolution of the cryo-EM maps reach an acceptable value. Activity assays, RALS and negative stain EM are repeated three times, which is sufficient given the robustness of the experiments.
Data exclusions	Cryo-EM particles that poorty aligned to produce final strucutres were not included as detemined by the accepted processing methods described in the paper and as reported in Extended Data Table 1. All replications in other experiments are successful.
Replication	Activity assay, RALS and negative stain EM assay were performed on 3 independent replicates. All replications are successful.
Randomization	Cryo-EM particles images were randomly assigned and compared for resolution determination by Fourier Shell Correlation (FSC). For other experiments, the samples are randomly allocated into experimental groups, by using the homogeneous protein and substrate solution.
Blinding	Blinding was not applicable to this study as there was no comparison groups.

Reporting for specific materials, systems and methods

We require information from authors about some types of materials, experimental systems and methods used in many studies. Here, indicate whether each material, system or method listed is relevant to your study. If you are not sure if a list item applies to your research, read the appropriate section before selecting a response.

Materials & experimental systems

n/a	Involved in the study
☒	☐ Antibodies
☒	☐ Eukaryotic cell lines
☒	☐ Palaeontology and archaeology
☒	☐ Animals and other organisms
☒	☐ Clinical data
☒	☐ Dual use research of concern
☒	☐ Plants

Methods

n/a	Involved in the study
☒	☐ ChIP-seq
☒	☐ Flow cytometry
☒	☐ MRI-based neuroimaging

Plants

Seed stocks	N/A
Novel plant genotypes	N/A
Authentication	N/A
